# An agonist antibody prefers relapsed AML for induction of cells that kill each other

**DOI:** 10.1038/s41598-019-40087-7

**Published:** 2019-03-05

**Authors:** Kyungmoo Yea, Teresa M. Jones, Dokyung Jung, Sanghee Shin, Britni M. Arlian, Kyung Ho Han, Zhao Zha, Minseok S. Kim, Yong-Seok Oh, Hongkai Zhang, Richard A. Lerner

**Affiliations:** 1 Department of New Biology, DGIST, Daegu, 42988 Republic of Korea; 2 Department of Chemistry, The Scripps Research Institute, La Jolla, CA 92037 USA; 3 Department of Molecular Medicine, School of Medicine, Kyungpook National University, Daegu, Republic of Korea; 4 Department of Molecular Medicine, The Scripps Research Institute, La Jolla, CA 92037 USA; 5 Department of Brain-Cognitive Science, DGIST, Daegu, 42988 Republic of Korea; 6 State Key Laboratory of Medicinal Chemical Biology, College of Life Sciences, Nankai University, Tianjin, China

## Abstract

Previously, we reported an agonist antibody to a cytokine receptor, Thrombopoietin receptor (TPOR) that effectively induces cytotoxic killer cells from precursor tumor cells isolated from newly diagnosed AML patients. Here, we show that the TPOR agonist antibody can induce even relapsed AML cells into killer cells more potently than newly diagnosed AML cells. After stimulation by the agonist antibody, these relapsed leukemic cells enter into a differentiation process of killer cells. The antibody-induced killer cells express, Granzyme B and Perforin that assault and kill other members of the AML cell population. Particularly, the agonist antibody showed potent efficacy on the AML xenograft model in mice using the NOD/LtSz-scid IL2Rγc null (NSG) mice. These results show that the TPOR agonist antibody that induces AML cells to kill each other is effective on both relapsed AML cells and *in vivo*. Therefore, this study suggests a new strategy for the treatment of cancer relapse after chemotherapy.

## Introduction

Tumor relapse with its attendant acquired cellular resistance to chemotherapy is the scourge of cancer treatment. Generally, once relapse occurs the prognosis is poor. There are classical reasons for resistance that include altered membrane transport, mutation of the target, drug degradation, or failure to promote apoptosis^1,2^. Recently, in addition to the classical models of resistance, much attention is being paid to cancer stem cells^3^. Arguably, the role of cancer stem cells in tumor relapse is best understood for acute myelogenous leukemia, largely because of the pioneering studies of Dick and colleagues^4^. These stem cells are thought to constitute a reservoir of tumor cells that are resistant to chemotherapy because of their capacity for self-renewal and relatively low rate of entry into the cell cycle^3^. Thus, when cancer relapses the tumor may be composed largely of stem cells because the process of chemotherapy selects for them. Here, we show that an agonist antibody that induces AML cells to kill each other is highly selective for relapsed cells. This occurs likely because the chemotherapy process selected for a population of stem cells that constitute the relapsed tumor and, in terms of induction of a new phenotype, stem cells are the preferred target for the agonist antibody.

Recently, we have isolated many agonist antibodies that induce differentiation in stem cells^5–11^. One of these is an agonist antibody (Clone name; 3D9) against TPOR that induces primary AML cells to differentiate into natural killer cells that, in a fratricidal process, kill one another^9,11^. This effect takes advantage of phenomena we termed receptor pleiotropism where agonist antibodies induce different cellular fates in stem cells than those induced by the natural agonists. This can occur even when the target of the natural agonist and the antibody are the same, thus the term receptor pleiotropism. In the case studied here an agonist antibody against the TPOR induced AML cells isolated from newly diagnosed patients to differentiate into killer cells^9^. These induced cells used their newly formed multiple elongated dendrites to capture neighboring cancer cells and kill them^9^. In addition to marked changes in morphology, the antibody induced expression of Perforin, Granzyme B and Interferon γ which are classical markers of killer cells. Neither the change in morphology nor expression of the proteins that are expressed in killer cells were seen in the absence of the agonist antibody. By contrast to its effects on AML cells this antibody to the TPOR induces normal CD34^+^ cells to differentiate into megakaryocytes and platelets *in vitro* and *in vivo* and no killer cells are seen^9,11^.

One criticism of our work could be couched in terms of medical need and the resistance of cells that recur after treatment to any therapy. Thus, in their armamentarium oncologists have plenty of drugs to eliminate some primary tumors and the real problem is when the tumor relapses. The cells in a relapsed tumor, by definition, have been selected to resist therapy. Some oncologists expected resistance to be a general property such that these relapsed cells would also be largely resistant to other therapeutic modalities including the agonist antibody. We, however, expected just the opposite and predicted an inversion from that which is usually seen in that the antibody would be more effective on relapsed tumors. The logic was that the recurrent cells are more stem cell like and stem cells are the very “substrate” of the agonist antibody. Actually, the stem cell phenotype is obligatory for induction. Here we show that, indeed, our therapeutic antibody shows an inversion from that seen in classical cancer therapy in that it kills recurrent tumor cells much more efficiently than cells of the primary tumor. Thus, there could be a new paradigm for cancer therapy where during chemotherapy there is an actual selection of cells that are sensitive to the next therapeutic modality and one needs to search for antibodies that selectively kill cell populations selected by chemotherapy.

## Results

### An antibody that potently induces differentiation of relapsed AML cells

AML is a hematological malignancy characterized by the presence of specific cell types and outcomes^12^. AML is associated with poor long-term survival, even when newer chemotherapeutic agents are used. Recent studies have reported that the AML relapse and resistance to conventional chemotherapies may originate from a small population, known as AML stem cells^4^. Given that we had generated an agonist antibody that induced differentiation of bone marrow stem cells, we wondered whether the responsiveness of a population of AML cells to the antibody could be changed after chemotherapy^9^. To compare the size of the stem cell populations between newly diagnosed and relapse, we used patient samples from 3 newly diagnosed and 3 relapsed AML patients. Two of the samples from the newly diagnosed AML patients were classified according to accepted standards as M1 (without maturation), and other was M2 (acute myeloblastic leukemia with maturation) whereas in the case of relapsed AML, one sample was classified as M1 and the other two were M2.

**Figure 1:**
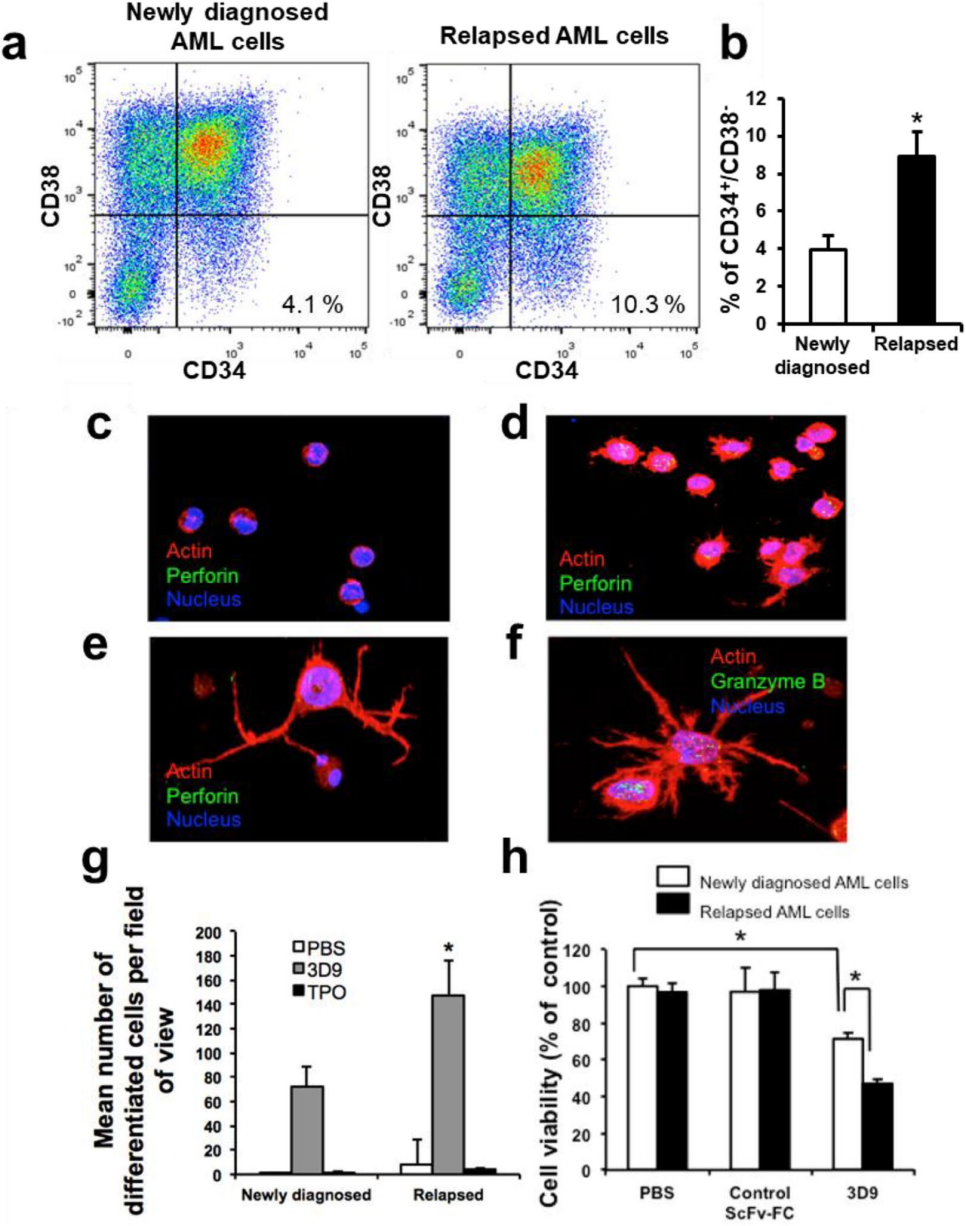
Analysis of differentiated killer cells from relapsed AML cells by agonist antibody. (**a**) Illustrates the FACS data. Both newly diagnosed AML cells and relapsed AML cells were labeled with anti-CD34 and anti-CD38 antibodies, which were conjugated with FITC or PE respectively and analyzed by FACS. (**b**) Represents mean % of CD34^+^/CD38^−^ population of newly diagnosed AML cells and relapsed AML cells from (**a**). Data are shown as mean ± SE (n = 3). Mann–Whitney test, *P < 0.05. (**c**,**d**) Illustrate morphology of AML cells after treatment with PBS or 10 μg/ml agonist antibody for 4 days. **(e**,** f**) Are the magnified images of fully differentiated cells in panel (d). The differentiated AML cells were labelled with fluorescent antibodies for Perforin or Granzyme B in **(c**,** d)**, **(e**) or **(f**) respectively and visualized by confocal microscopy. Nuclei were labelled with Hoechst 33342 (blue). F-actin is stained with Rhodamin-phalloidin (red). (**g**) Shows mean number of differentiated cells from newly diagnosed or relapsed AML cells by PBS, agonist antibody or TPO. Differentiated cells were counted from 5 microscopic areas, which were randomly chosen. 3 different AML cells of newly diagnosed or relapsed AML patients were used respectively. Relapsed AML cells are converted to killer cells about 2 times more than newly diagnosed AML cells by agonist antibody. Data are shown as mean ± SE (n = 3). 2-way ANOVA with Bonferroni’s multiple comparison test, *P < 0.05. (**h**) The AML cells were stained with Calcein-AM and co-cultured with differentiated cells or undifferentiated cells. The antibody-induced killers are obtained from both newly diagnosed AML cells or relapsed AML cells. Cells were co-cultured for 24 hr at 37 °C. After co-culture of the cells, dead cells were stained with PI. The percentage of dead cells was analyzed by flow cytometry. Data are shown as mean ± SE (n = 5). 2-way ANOVA with Bonferroni’s multiple comparison test, *P < 0.05.

It is known that some AML stem cells are CD34^+^/CD38^−^^13,14^. To quantitate this population of stem cells in the AML samples, we carried out Fluorescence-activated cell sorting (FACS) analysis after fluorescent cell surface labeling of CD34 and CD38 markers. According to the FACS analysis, in the three cases of relapsed AML the CD34^+^/CD38^−^ population of cells was about 2.5 fold higher than that of newly diagnosed AML cells (Fig. 1a, b). Based on this observation, we hypothesized that the relapsed AML cell population may be more susceptible to agonist antibody-induced killer cell differentiation because, as noted above, the stem cell phenotype is an obligatory component of the mechanism of action of the antibody. Thus, we next tested whether the agonist antibody can induce killer cell phenotypes even in relapsed AML cells. First, we checked whether TPOR is expressed in several AML subsets and found that TPOR is abundantly expressed both in newly diagnosed and relapsed AML cells (Supplementary Fig. S1). When the relapsed AML cells were incubated with the agonist antibody, they were also attached to the culture dish and formed multiple short filopodia (Fig. 1d). Nearly no cells adhered to dish in the absence of the agonist antibody (Fig. 1c). In the course of several experiments we found that the majority of cells from relapsed AML patients fully differentiate into killer cells after 4 days (Fig. 1d). Figure 1e, f represent the magnified images of fully differentiated cells in Fig. 1d. During this time the short filopodia from cells of the initial phase were further elongated and the cells began to express Perforin and Granzyme B abundantly (Fig. 1d–f, Supplementary Fig. S2).

### The Agonist antibody preferentially causes relapsed AML cells to be killed

To gain quantitative information about the responsiveness of AML cells against the agonist antibody, we quantitated the number of cells induced to become killer cells in the relapsed versus starting AML population. After 6 days induction, relapsed AML cells are converted to killer cells about 2 times more than newly diagnosed AML cells (Fig. 1g). To determine if the increase in the number of killer cells affects the killing potential of the population, we measured the cytotoxic activity of these antibody-induced killer cells. Induced killer cells from a newly diagnosed AML cell population reduced the cell viability by 30% in 24 hr. By contrast, 50% of cells from the relapsed AML population were killed (Fig. 1h). These data again suggest that the relapsed AML cell population is more susceptible to induction of differentiation by the agonist antibody.

### Single cell studies

**Figure 2:**
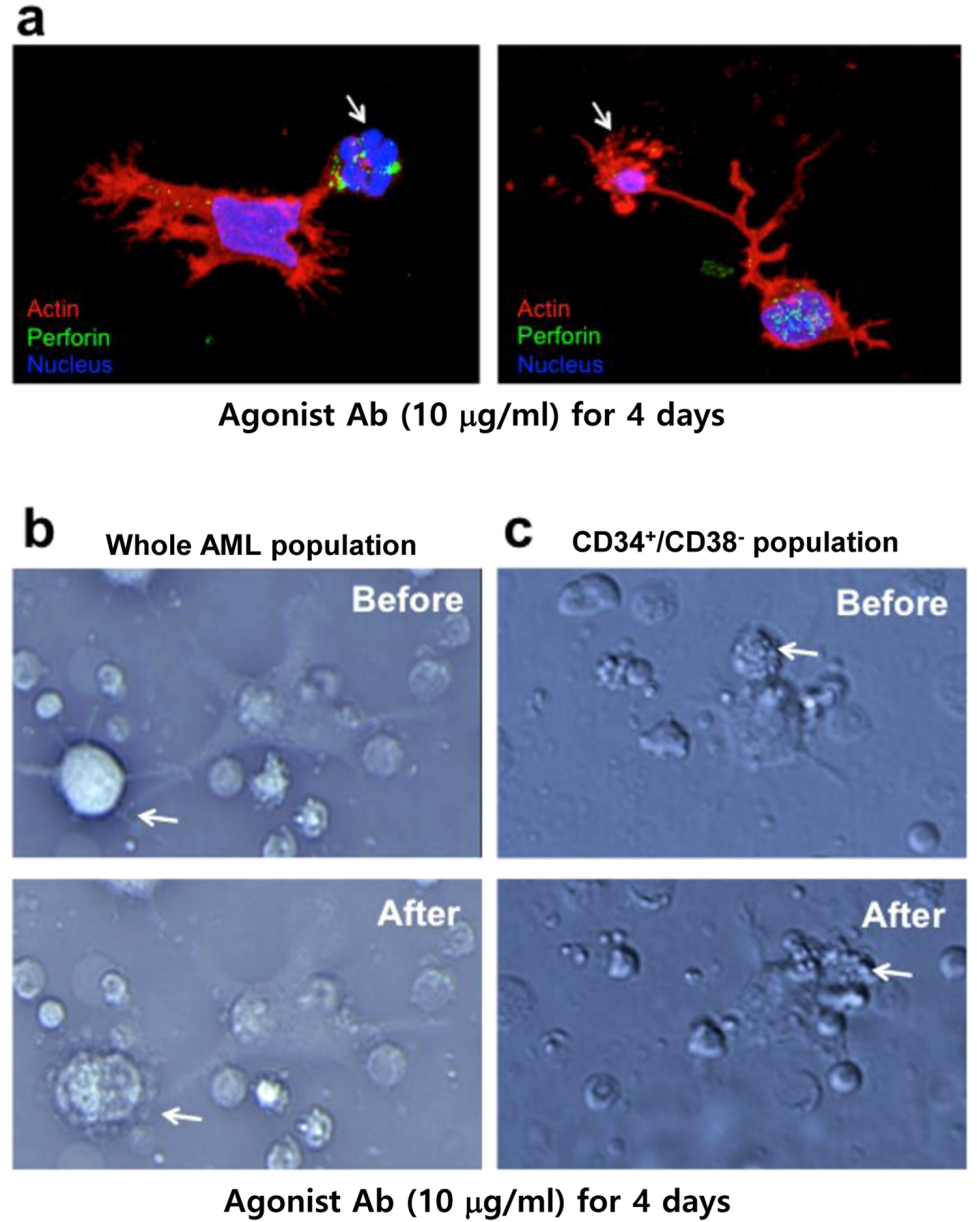
Single cell analysis of AML cells. (**a**) Represents images of antibody-induced killer cells capturing target AML cells by confocal microscopy. The arrow shows capturing of target AML cells by filopodia of an agonist antibody-induced killer cell. The antibody-induced differentiated cells were labelled with Perforin. The blue color represents nucleus. F-actin is labeled with Rhodamin-phalloidin (red). (**b**) Whole AML population was treated with agonist antibody (10 μg/ml) for 4 days, and then incubated undifferentiated AML cells. Upper panel shows the initial phase of the target cell killing by the killer cell. Lower panel shows the later phase of target cell killing by the killer cell. (**c**) CD34^+^/CD38^−^ population of AML cells were incubated with agonist antibody (10 μg/ml) for 4 days, and then incubated undifferentiated AML cells. The target cell killing by the killer cell was monitored as **(a)** White arrow indicates the attacked target AML cells by killer cells.

Since all previous studies were carried out on cell populations, we wished to observe the killing process on relapsed AML cells at the single cell level. We observed that initially many more cells from the relapsed as compared to the newly diagnosed AML population attach to the dish. These attached cells then develop long filopodia that capture the target cells as yet undifferentiated AML cells (Fig. 2a). Clearly these differentiating cells arise from cells that have a very different morphology from the starting AML cells. To ensure that the filopodia based cell attachment to target cells is actually followed by death of the target cells we studied the process using time lapsed microscopy. We showed that attachment of these actively moving long filopodia from killer cells is followed by explosive rupture of the captured target cells (Fig. 2b and Supplementary Movie 1). In addition to the killer cells derived from total relapsed AML cells, we also observed that the cytotoxic activity of the killer from CD34^+^/CD38^−^ AML stem cells (Fig. 2c and Supplementary Movie 2).

### TPOR signal activation by agonist antibody

**Figure 3:**
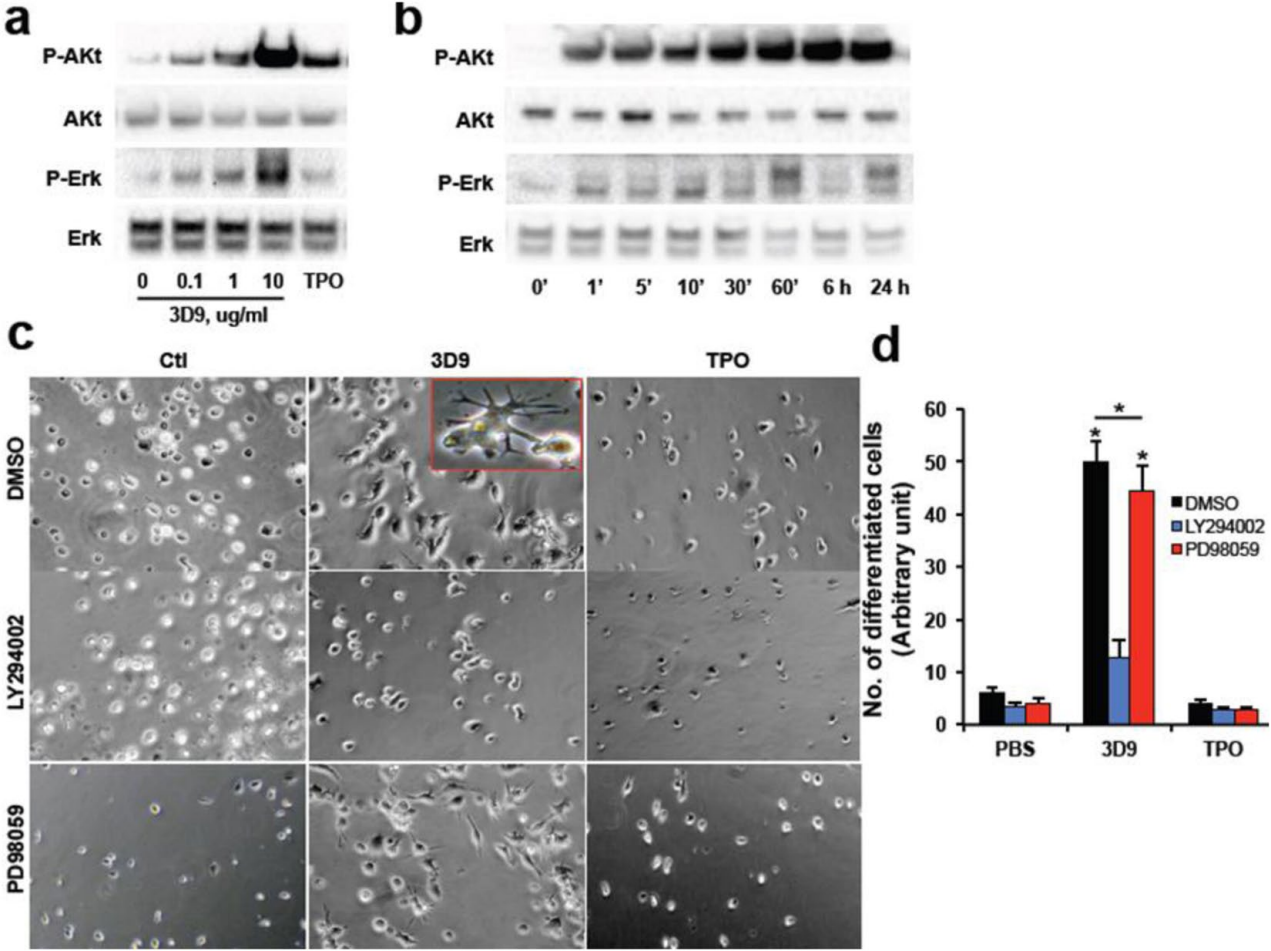
TPOR signal activation by the agonist antibody in relapsed AML cells. (**a**) After incubation with various doses of the agonist antibody or recombinant TPO (10 ng/ml) for 1 hr, the phosphorylation level of AKT and ERK was investigated by western blotting. **(b)** The activation of TPOR signaling was analyzed at various time points after the agonist antibody or TPO treatment. **(c)** Relapsed AML cells after incubation with PBS, the agonist antibody (10 μg/ml) or recombinant TPO (10 ng/ml) for 4 days in the presence of the inhibitors of PI3K (LY294002) or MAPK (PD98059). **(d)** The numbers of differentiated cell were analyzed in eight randomly chosen microscopic areas. Significant differences (*P < 0.05) (n = 3) were analyzed by 2-way ANOVA with Bonferroni’s multiple comparison test.

We analyzed the activation of signal transduction after agonist antibody treatment in the relapsed AML cells. According to our previous study, the agonist antibody induced potent activation of AKT/Protein kinase B, and Extracellular signal-regulated kinase (ERK) phosphorylation in the newly diagnosed AML cell population. Similarly, in relapsed AML cells the antibody again potently activates AKT and ERK compared to induction with natural TPO (Fig. 3a, b). To analyze the signaling pathways required for killer cell induction by agonist antibody, relapsed AML cells were co-treated with agonist antibody and chemical inhibitors of Phosphatidylinositol-3-kinase (PI-3K) or Mitogen-activated protein kinase (MAPK) signaling pathways. Intriguingly, antibody-dependent killer cell induction was inhibited significantly by the inhibitor of PI-3K that are upstream signal of AKT. However, the specific inhibitor of MAPK did not influence the differentiation of cells (Fig. 3c, d). These observations imply that one of the two main signaling pathways of the TPOR (PI-3K) is critical for the antibody-induced killer cell differentiation from AML cells.

### The agonist antibody protects mice in AML xenograft models

**Figure 4:**
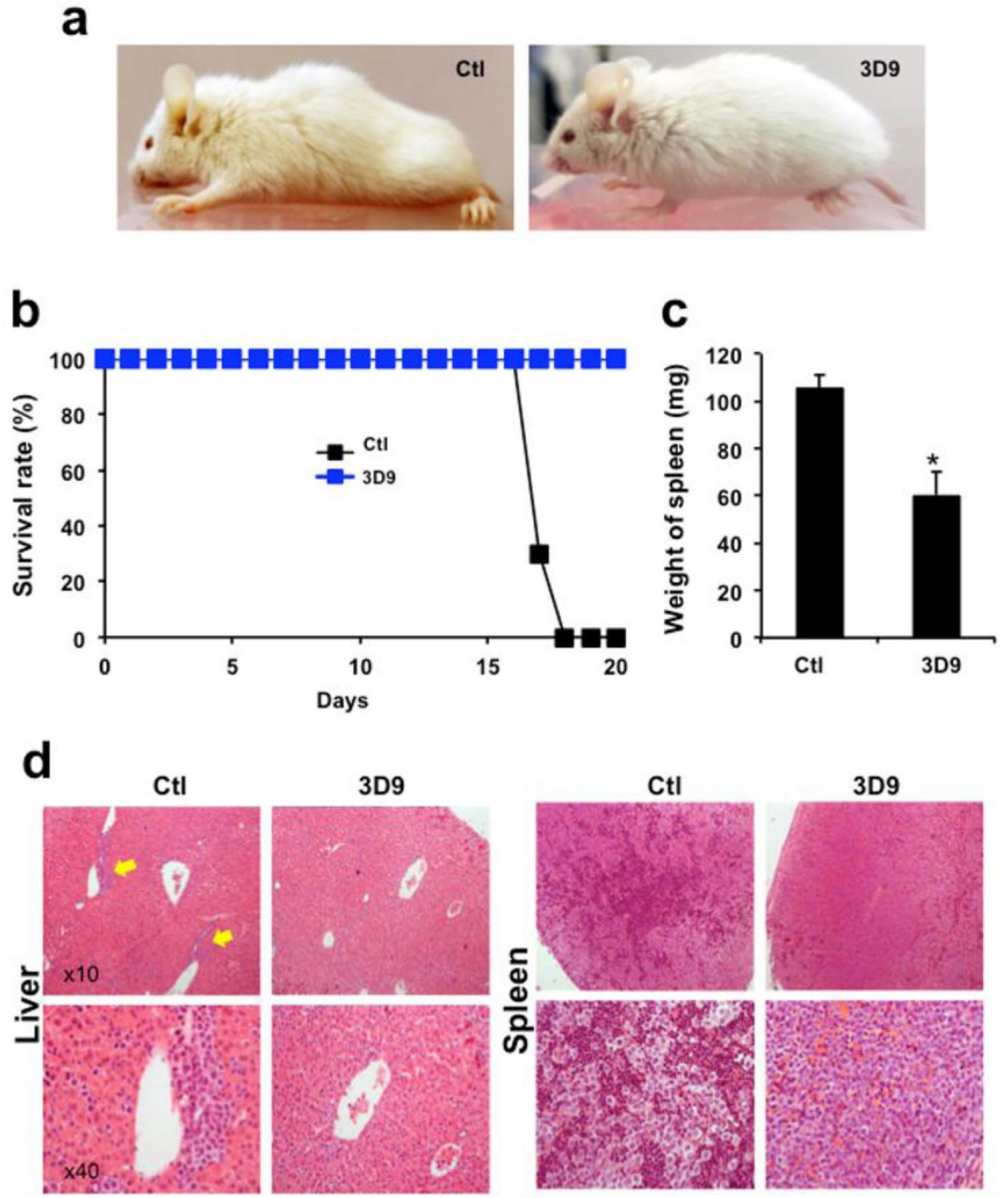
Evaluation of the antibody efficacy in the leukemic xenograft model. (**a**) Shows the representative appearance of PBS or antibody injected xenograft mice after 10 days. **(b)** Survival of mice was monitored for 20 days after injection of PBS or antibody (10 mg/kg). **(c)** Whole spleens were isolated from mice 16 days after PBS or antibody injection and weighed. Data are shown as mean ± SE (n = 5). Mann–Whitney test, *P < 0.05. **(d)** Whole livers and spleens were isolated from mice 16 days after PBS or antibody injection and dissected for histological analysis. All tissue sections were fixed and stained with H&E.

To test the efficacy of the agonist antibody *in vivo*, we first established an AML xenograft model in mice using the NOD/LtSz-scid IL2Rγc null (NSG) mice. We studied 6 experimental and 6 control mice. The 4-week-old NSG mice were sub-lethally irradiated 1 day before *i.v*. injection of 2 million AML cells. Then the mice were treated with the agonist antibody (10 mg/kg) or PBS every other day. The behavior and condition of the mice were routinely analyzed. After 10 days, mice in the non-treated control group began to show evident disease related properties such as ruffled coat, hunched back, weakness and reduced motility but mice in the group treated with antibody did not (Fig. 4a). By 18 days all mice in the untreated group died but all the mice treated with antibody were still healthy (Fig. 4b). One of the key findings in AML is the enlargement of organs such as spleen and liver. After sacrificing the mice, we compared the weight of spleen from the control group to the antibody treated group. The spleen weight of antibody treated mice was 50% lower than the untreated control group and comparable to that of normal mice (Fig. 4c). Furthermore, we analyzed the sections of liver and spleen by H&E and immunohistochemistry to determine if the engrafted AML cells infiltrated into these tissues. The liver and spleen were heavily infiltrated by AML cells (Fig. 4d). In the liver the infiltrating AML cells localized primarily around portal veins. Importantly, no infiltrating AML cells were observed in mice treated with antibody. These results suggest that the cytotoxicity of the antibody-induced killer cells extends to tumor cells *in vivo*.

## Discussion

The main purpose of this paper is to suggest a new protocol for the treatment of cancer relapse after chemotherapy. It is based on the recent discovery of receptor pleiotropism where one can select many agonist antibodies to known receptors on stem cells that cause them to differentiate along pathways and display phenotypes that differ from the response of these same receptors to their physiological agonists^6,8,15^. The central idea is that when cancer relapses after treatment the malignant cell population is enriched for stem cells that are the very cellular substrate for most agonist antibodies. Indeed, stem cells are not simply a preferred but rather are an obligatory target for agonist antibodies.

The concept that control of AML remissions lies in elimination of stem cells is by no means unique to us^9^. The difference is that we suggest an immuno-therapy that relies on inducing differentiation in recurrent AML cells rather than killing them. There are many outcomes that one might wish to happen after an agonist antibody induces an alternative phenotype in cancer stem cells. These range from increased sensitivity to chemotherapy to loss of malignancy in the newly differentiated cells. In the present study, we have an additional advantage because treatment with the agonist antibody causes the relapsed AML population to differentiate into killer cells. This is likely to be a special, albeit highly desirable, outcome pejorative to AML stem cells and probably is not generalizable to other cancers. Nevertheless, it would be almost as good if one could select an antibody where the induced differentiation led to loss of the malignant phenotype or increased sensitivity to chemotherapy.

From a practical point of view, all the reagents to test this concept are in place. In principle, one would select from large combinatorial libraries of agonist antibodies that induce phenotypic changes in cancers that recur after chemotherapy. In this scenario, since the chemotherapy selects for stem cells from the original AML population, it is “creating” the substrate for the agonist antibodies. However, in these early times it’s not clear whether there are antibody inducible stem cells from different cancers and whether the induced cells are similar even within a given tumor type.

We tested M1 and M2 AML classes from newly diagnosed and relapsed AML patients. According to our experiments, there were no significant difference between M1 and M2 from newly diagnosed or relapsed AML cells regarding differentiation potential and cytotoxicity of killer cells induced by agonist antibody. Furthermore, the CD34^+^/CD38^−^ population also was similar between M1 and M2 AML cells. In the future, it will be intriguing to compare the activity of agonist antibody in other AML classes such as M3 to M7 AML cells.

Although the antibody efficiently inhibited the progress of AML in the xenograft model, still it is not clear whether killer cells are induced *in vivo* by agonist antibody as well. Further characterization of killer cells from *in vivo* samples by using immunohistochemistry analysis may enhance the elucidation of the *in vivo* mechanism of antibody action.

A remaining question is what is the definition of a stem cell in the context of agonist antibody treatment? The simple answer is that a stem cell is a cell that is induced to differentiate by the agonist antibody regardless of other parameters. This is an operational definition and may differ from that determined by other parameters such as cell surface markers. To this end, we expect from our morphological studies that, in terms of this definition, the inducible AML stem cell population studied here is much larger than that simply defined by CD34^+^ and CD38^+^ cells.

## Methods

### Culture of AML cells

Human AML cells were isolated from patients and frozen at −80 °C (ABM010, AllCells). The informed consent was obtained from all patients. AML cells were grown in StemSpan serum-free media (Stemcell technologies) containing streptomycin and penicillin. Chemical inhibitors of PI-3K (LY294002) and MAPK (PD98059) were purchased from Sigma-Aldrich. All procedures were carried out in accordance with the relevant guidelines and regulations. All experimental protocols were approved by the Institutional Animal Care and User Ethical Committee of the Scripps Research Institute.

### Expression and purification of 3D9, the agonist antibody

The sequence of heavy chain complementarity region (H-CDR3) and light chain complementarity region (L-CDR3) of 3D9 clone are CARGGLLRPFDYWG and QQSNSFPWT respectively. The agonist antibody DNA was transfected into 293 F cells using Lipofectamine 2000 (Invitrogen). Antibody proteins from culture supernatants were purified using ÄKTAxpress purifier (HiTrap Protein G affinity HP columns). The elution buffer was exchanged to Dulbecco’s PBS (pH 7.4) containing protease inhibitor cocktails (Sigma- Aldrich).

### Immunocytochemistry

Cells were fixed at room temperature (RT) for 15 min in 4% paraformaldehyde solution, blocked by 5% goat serum and labelled with primary antibodies, followed by fluorescent secondary antibody staining. Hoechst 3342 is from Cell signaling, Rhodamine-phalloidin is from Life technologies. All antibodies were incubated for 1 hr at RT. After washing 3 times with PBS for 5 min, images were analyzed using a confocal microscope.

### Flow cytometry

AML cells were incubated with Isotype control, CD34-FITC (clone 4H11) or CD38-PE (clone HB7) antibodies (Invitrogen) for 1 hr. After removal of labeling antibodies, cells were resuspended in ice-cold FACS buffer and analyzed by LSRII flow cytometer (Becton Dickinson).

### Cytotoxicity assay

The target cancer cells were stained with 2 μg/ml Calcein-AM (Life technologies) for 20 min. Target cells were washed, and resuspended at 1 × 10^6^ cells/ml in culture media. Differentiated killer cells were resuspended at 2 × 10^6^ cells/ml in culture media. Then, each resuspended cells were co-cultured for 24 hr. After co-culture, differentiated AML cells and the target cells were labelled with PI (Life technologies) to determine dead cells. For quantitative analysis, labelled cells were analyzed with a LSRII flow cytometer (Becton Dickinson).

### Western blotting

To prepare whole cell lysates, AML cells were washed with PBS twice and then lysed in lysis buffer (10% glycerol, 50 mM NaF, 150 mM NaCl, 1 mM Na_3_VO_4_, 1% Triton X-100, 50 mM HEPES, pH 7.2). The cell lysates were then centrifuged at 12,000 rpm for 5 min at 4 °C to discard aggregates, and the soluble fractions were denatured in LDS sample buffer (5 min at 95 °C, Thermo Fisher Scientific) and then separated by gel electrophoresis (SDS-PAGE). The proteins were electro-transferred to nitrocellulose membranes using the iBlot 2 Gel transfer device system (Invitrogen) and blocked in 5% BSA for 30 min before being incubated with primary antibodies for overnight at 4 °C. After washing the nitrocellulose membranes with PBST for 1 hr, the blots were incubated with horseradish peroxidase-conjugated anti-mouse or anti-rabbit secondary antibody for 1 hr. Then, the membranes were washed with PBST for 30 min and developed by ECL (GE). Anti-ERK1/2 and anti-pERK1/2 (Thr-202/Tyr-204), anti-AKT and anti-pAKT (Thr-308) antibodies were obtained from Cell signaling.

### Time-lapse live-cell imaging

Antibody-induced differentiated cells from total population of relapsed AML cells or CD34^+^/CD38^−^ relapsed AML cells were co-cultured with undifferentiated target cells in a 96-well plate at 37 °C. The ratio between differentiated cells and target cells were 2:1. Cell images are monitored under bright-field for 6 hr using the In Cell Analyzer 6000 (GE). Cells were cultured in a 5% CO_2_ condition.

### Xenotransplantation of human leukemic cells

Animals were used in accordance with a protocol reviewed and approved by the Institutional Animal Care and User Ethical Committee of the Scripps Research Institute (Approval #:06-0325). NSG mice were purchased from The Jackson Laboratory. Mice were housed in sterile conditions and fed with irradiated food. 4 weeks old mice were sublethally irradiated with 250 cGy of total body irradiation 24 hr before injection of AML cells. AML cells were washed twice in DPBS and suspended in 200 μl of PBS at a concentration of 2 million cells per mouse for *i.v*. injection. PBS or agonist antibody (5 mg/kg) was administrated twice per week. Daily monitoring of mice for AML symptoms determined the day of animal euthanization. All methods were carried out in accordance with the relevant guidelines and regulations.

### Statistical analysis

All data are described as mean ^+^/^−^ SE. Statistical analysis was performed using GraphPad prism software (version 7.0, US). Differences between the two groups in terms of continuous variables were analyzed by using the Mann–Whitney test or 2-way ANOVA with Bonferroni’s multiple comparison test. Statistical tests used to determine significance are indicated in the figure legends.

## Supplementary information

Supplementary info

Video-1

Video-2
