# Supplementary material for: An agonist antibody prefers relapsed AML for induction of cells that kill each other
Source: Sci Rep. 2019 Mar 5;9:3494. doi: 10.1038/s41598-019-40087-7 (PMC6401169; doi:10.1038/s41598-019-40087-7)
Supplement: Supplementary file 1 — Supplementary info [file 41598_2019_40087_MOESM1_ESM.docx]

**An agonist antibody prefers relapsed AML for induction of cells that kill each other**

Authors:

Kyungmoo Yea, Teresa M. Jones, Dokyung Jung, Sanghee Shin, Britni M. Arlian, Kyung Ho Han, Zhao Zha, Minseok S. Kim, Yong-Seok Oh, Hongkai Zhang, Richard A. Lerner

**Supplemental Figure Legends**

**Figure. S1. TPOR expression in AML cells.** TPOR expression was detected in M1 and M2 AML cells of both newly diagnosed and relapsed AML patients.

**Figure. S2. The immunocytochemistry of differentiated AML cells.** a. F-actin is labeled by rhodamin-phalloidin (red). b. The differentiated cells were stained for actin Perforin, Granzyme B were stained. Nucleus was stained by Hoechst 33342 (blue).

**Movie 1.** Whole AML population was treated with agonist antibody (10 μg/ml) for 4 days, and then undifferentiated AML cells were added to whole AML cell culture. The whole process of target cells killing by killer cells was recorded for 6 hr, under 5% CO_2_ condition.

**Movie 2.** CD34^+^/CD38^-^ population of AML cells were incubated with agonist antibody (10 μg/ml) for 4 days, and then incubated undifferentiated AML cells. The whole process of target cells killing by killer cells was recorded for 6 hr, under 5% CO_2_ condition.

**Figure. S1**

**
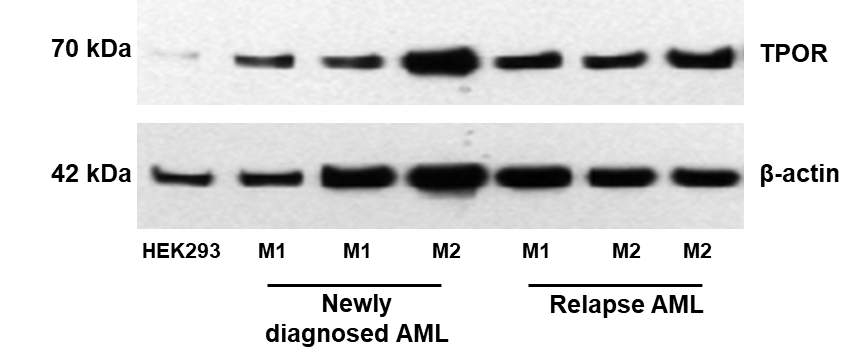
**

**Figure. S2**

**
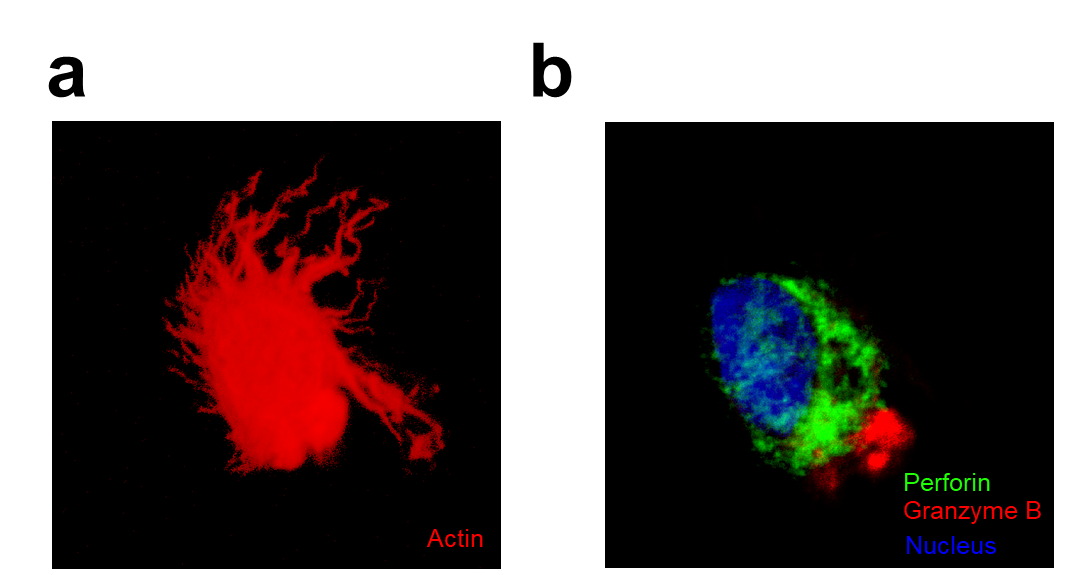
**
